# Evidence That the Anti-Inflammatory Effect of Rubiadin-1-methyl Ether Has an Immunomodulatory Context

**DOI:** 10.1155/2019/6474168

**Published:** 2019-11-03

**Authors:** Eduarda Talita Bramorski Mohr, Marcus Vinicius Pereira dos Santos Nascimento, Júlia Salvan da Rosa, Guilherme Nicácio Vieira, Iara Fabricia Kretzer, Louis Pergaud Sandjo, Eduardo Monguilhott Dalmarco

**Affiliations:** ^1^Department of Clinical Analysis, Center of Health Sciences, Federal University of Santa Catarina, Campus Universitário-Trindade, 88040-970 Florianópolis, SC, Brazil; ^2^Department of Pharmaceutical Sciences, Center of Health Sciences, Federal University of Santa Catarina, Campus Universitário-Trindade, 88040-970 Florianópolis, SC, Brazil

## Abstract

**Background:**

In spite of the latest therapeutic developments, no effective treatments for handling critical conditions such as acute lung injuries have yet been found. Such conditions, which may result from lung infections, sepsis, multiple trauma, or shock, represent a significant challenge in intensive care medicine. Seeking ways to better deal with this challenge, the scientific community has recently devoted much attention to small molecules derived from natural products with anti-inflammatory and immunomodulatory effects.

**Aims:**

In this context, we investigated the anti-inflammatory effect of Rubiadin-1-methyl ether isolated from *Pentas schimperi*, using an *in vitro* model of RAW 264.7 macrophages induced by LPS and an *in vivo* model of acute lung injury (ALI) induced by LPS.

**Methods:**

The macrophages were pretreated with the compound and induced by LPS (1 *μ*g/mL). After 24 h, using the supernatant, we evaluated the cytotoxicity, NOx, and IL-6, IL-1*β*, and TNF-*α* levels, as well as the effect of the compound on macrophage apoptosis. Next, the compound was administered in mice with acute lung injury (ALI) induced by LPS (5 mg/kg), and the pro- and anti-inflammatory parameters were analyzed after 12 h using the bronchoalveolar lavage fluid (BALF).

**Results:**

Rubiadin-1-methyl ether was able to inhibit the pro-inflammatory parameters studied in the *in vitro* assays (NOx, IL-6, and IL-1*β*) and, at the same time, increased the macrophage apoptosis rate. In the *in vivo* experiments, this compound was capable of decreasing leukocyte infiltration; fluid leakage; NOx; IL-6, IL-12p70, IFN-*γ*, TNF-*α*, and MCP-1 levels; and MPO activity. In addition, Rubiadin-1-methyl ether increased the IL-10 levels in the bronchoalveolar lavage fluid (BALF).

**Conclusions:**

These findings support the evidence that Rubiadin-1-methyl ether has important anti-inflammatory activity, with evidence of an immunomodulatory effect.

## 1. Introduction

Anthraquinones are an important class of synthetic and natural compounds with a wide and diverse range of actions. In addition to their use as dyes, they present highly pharmacological diversity with proven laxative, anticancer, antiarrhythmic, antibacterial, antifungal, antiviral, and anti-inflammatory activities [1]. The genus Pentas belongs to the Rubiaceae family. It is known for its anthraquinone compounds and is routinely used in traditional medicine, mainly in the African continent, to treat dysmenorrhea, headache, and pyrexia [2, 3]. Rubiadin-1-methyl ether is an anthraquinone compound easily isolated from the Pentas species. However, there are a few reports that demonstrate its biological effects [4]. Thus, despite the anti-inflammation-proved effects of anthraquinones, we decided to evaluate this potential effect on Rubiadin-1-methyl ether compound [4, 5].

The inflammatory reaction is a defensive response against factors that disrupt host homeostasis. This phenomenon is modulated through a complex system of factors. After the initial trigger, there are production and release of protein-based and lipid mediators, which coordinate and increase the blood flow and permeability of the blood capillaries, inducing the movement of leukocytes to the initial stimulus [6]. Among the leukocytes involved, macrophages represent a pivotal role, due to their ability to recognize pathogen-associated molecular patterns (PAMPs) such as lipopolysaccharide (LPS), one of the main molecules responsible for the development of acute lung injury [7]. Despite recent therapeutic technologies, there is a lack of adequate treatment for this condition, which represents an important challenge in intensive care medicine. Recently, research has focused on small molecules derived from natural products with anti-inflammatory and immunomodulatory effects, since this condition is the result of an intricate and complex inflammatory response [8]. In this context, we evaluated the anti-inflammatory properties of Rubiadin-1-methyl ether, investigating its effects on cytotoxicity; nitric oxide (NOx) inhibition; IL-6, IL-1*β*, and TNF-*α* production; and the influence of this compound under macrophage apoptosis, using an *in vitro* model. We also performed an evaluation of Rubiadin-1-methyl ether on the leukocyte migration, fluid leakage, MPO activity, NOx, and pro- and anti-inflammatory cytokine (IL-6, IL-10, IL-12p70, MCP-1, INF-*γ*, and TNF-*α*) production, using an *in vivo* model.

## 2. Materials and Methods

### 2.1. Compound

**Figure 1:**
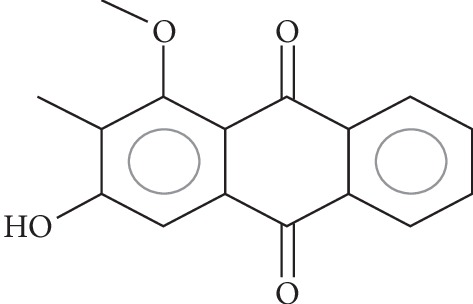
Chemical structure of the studied compound Rubiadin-1-methyl ether.

The compound was extracted and isolated by our research group from the roots of *Pentas schimperi*. Briefly, dried roots were used to extract Rubiadin-1-methyl ether in methanol (3 × 6 L). This solution was conditioned at room temperature for 72 hours to produce a crude extract after filtration and vacuum evaporation. The extract was subjected to column chromatography to isolate Rubiadin-1-methyl ether, according to the methodology already published [9] (Figure 1).

For the *in vitro* experiments, Rubiadin-1-methyl ether was dissolved in 1% dimethyl sulfoxide (DMSO), aliquoted, and stored at −20°C until the moment of the experiments, when it was properly dissolved in cell medium. During the *in vivo* assays, the compound was dissolved in a solution of sterile saline (0.9% NaCl), polysorbate-20 (5%, Tween-20), and dimethyl sulfoxide (5%, DMSO). The solutions were prepared on the same days as the experiments.

### 2.2. Reagents and Drugs

The following drugs and reagents used were obtained from BD Biosciences (San Diego, California, USA): IL-1*β* ELISA kit; FITC annexin V apoptosis detection Kit I, cytometric bead array-CBA mouse inflammation kit, albumin (bovine serum), and Folin and Ciocalteu's phenol reagent; from BioTech (São Paulo, São Paulo, Brazil): hydrogen peroxide 30%; from Gibco (Grand Island, New York, USA): Dulbecco's modified Eagle's medium (DMEM), fetal bovine serum (FBS), penicillin-streptomycin (10,000 U/mL), Versene® (2 g EDTA, Na_4_), and Trypan blue dye; from LaborClin (Pinhais, Paraná, Brazil): phosphate-buffered saline; from LabSynth (Diadema, São Paulo, Brazil): ethanol, trisodium citrate, sodium carbonate, copper sulfate, double tartrate Na/K, and sodium hydroxide; from Newprov (Pinhais, Paraná, Brazil): Panoptic® dye; from Peprotech (Rocky Hill, New Jersey, USA): mouse TNF-*α* and IL-6 ELISA kits; from Rio de Janeiro Cell Bank (Rio de Janeiro, Rio de Janeiro, Brazil): RAW 264.7 macrophages; from Sigma-Aldrich Co. (St. Louis, Missouri, USA): lipopolysaccharide 0111:B4 (*E. coli*), Dexamethasone (minimum 98% HPLC), Paclitaxel (minimum 95% HPLC), 3-(4,5-dimethylthiazol-2-yl)-2,5-diphenyltetrazolium bromide (MTT), hydrogen peroxide, hexadecyltrimethyl ammonium bromide, o-dianisidine % 2HCl (3,3′dimethoxybenzidine), *α*-naphthylethylenediamide % 2HCl, phenol, sodium azide, sodium dodecyl sulfate, sodium hypochlorite, sodium nitroprusside, sulfanilamide, and vanadium chloride (III); from Syntec (Hortolândia, São Paulo, Brazil): xylazine hydrochloride (2%) and ketamine hydrochloride (10%); from Vetec (Rio de Janeiro, Rio de Janeiro, Brazil): dimethyl sulfoxide, polysorbate-20 (5%), sodium hydrogen phosphate, and zinc sulfate. The other reagents used and nonlisted were obtained from alternative commercial sources.

### 2.3. In Vitro Experiments

#### 2.3.1. Cell Culture

RAW 264.7 murine macrophage cells were purchased from the Rio de Janeiro Cell Bank. The cells were maintained at 37°C in a 5% CO_2_ humidified atmosphere and cultured in Dulbecco's modified Eagle medium (DMEM), supplemented with 10% fetal bovine serum (FBS) and 1% antibiotics (100 U/mL penicillin, 100 *μ*g/mL streptomycin). All experiments were conducted after verification of viable cells by the Trypan blue technique. The experiments were conducted between the 3th and 8th passages, and all *in vitro* assays were performed in triplicate and repeated on different days (*n* = 3/group).

#### 2.3.2. Cell Viability (Cytotoxicity)

The compound cytotoxicity effect was evaluated by the MTT assay, already described [10]. The viability was measured after treatment with Rubiadin-1-methyl ether. For this, RAW 264.7 cells (5 × 10^4^ cells/well) were seeded in 96-well plates and incubated at 37°C for 24 h until complete adherence and confluence (in a 5% CO_2_ humidified atmosphere). The compound was tested at doses of 1 to 300 *μ*M, diluted in 1% DMSO, and incubated for more 24 h. Then the culture medium was replaced by MTT solution (5 mg/mL), followed by an incubation time of 2 h. A control group was performed in these identical conditions, using the same amount of DMSO, and cell viability was considered 100%. The MTT solution was removed, and the crystals were resuspended in DMSO. The results were evaluated by an ELISA reader, using a 450 nm wavelength.

Based on this test, it was possible to determine the CC_10_ of Rubiadin-1-methyl ether. The CC_10_ concentration represents the minimum necessary dose able to kill 10% of the cell line used [11] and was calculated through nonlinear regression analysis of the logarithm of concentration as a function of the normalized response (percentage of cell viability), using the software Prism 7.0 (GraphPad Software, La Jolla, CA, USA).

#### 2.3.3. Cell Inflammation Assay

To establish an inflammatory condition, RAW 264.7 macrophages were stimulated with *E. coli* lipopolysaccharide (LPS 1 *μ*g/mL). For this procedure, cells were seeded in 96-well plates and incubated until complete full adherence and confluence (37°C for 24 h; in a 5% CO_2_ humidified atmosphere). Next, cells were pretreated (0.5 h) according to the division groups, with subsequent induction by LPS (1 *μ*g/mL). After 24 h, the supernatants were collected and used to measure nitrite/nitrate (NOx) metabolite production and proinflammatory cytokine secretion (IL-6, IL-1*β*, and TNF-*α*).

The cell division groups were as follows: Blank control (B), represented by noninflamed cells that were only pretreated with vehicle (DMSO 1%); negative control (LPS), characterized by inflamed LPS cells and pretreated with vehicle; positive control (Dexa), with cells pretreated with Dexamethasone (7 *μ*M)—a reference anti-inflammatory drug; and the treatment groups (Rub), represented by macrophages pretreated with the CC_10_ of Rubiadin-1-methyl ether (*n* = 3/group).

#### 2.3.4. Measurement of Nitric Oxide Metabolites

NO production was measured indirectly using the supernatant to quantify the formation of its metabolites nitrate (NO_3_^−^) and nitrite (NO_2_^−^), through the Griess reaction [12]. The supernatant was collected 24 h after stimulation by LPS (1 *μ*g/mL), and the aliquots were mixed with an equal volume of Griess reagent. After incubation for 10 min, at room temperature, the results were determined by an ELISA plate reader at 540 nm. The absorbance was measured by interpolation from the nitrite standard curve (0–100 *μ*M), and the results were expressed in *μ*M.

#### 2.3.5. Quantification of Proinflammatory Cytokines (IL-6, IL-1*β*, and TNF-*α*)

The cytokines interleukin 6 (IL-6), interleukin 1 beta (IL-1*β*) and tumor necrosis factor alpha (TNF-*α*) present in the culture medium (supernatant) were measured by commercial kits, using the enzyme-linked immunosorbent assay (ELISA), following the manufacturer's instructions. Kits for IL-6 and TNF-*α* were purchased from Peprotech (Rocky Hill, United States of America), while the kit used for IL-1*β* was purchased from BD Biosciences (San Diego, California, USA). The levels of cytokines were estimated by interpolation from a standard curve by colorimetric measurements at 450 nm in an ELISA plate reader. The results were expressed in pg/mL.

#### 2.3.6. Determination of Macrophage Apoptosis

For this experiment, macrophages were seeded in 24-well plates and pretreated according to the division groups, with subsequent induction with LPS (1 *μ*g/mL) followed by incubation of 0.5 h. After 24 h, cells were scraped with Versene® (0.2 g EDTA, Na_4_) and centrifuged with DMEM (900 x g, 5 min at 4°C). The groups were divided into the following: Blank control (B), represented by noninflamed cells only pretreated with vehicle (DMSO 1%); negative control (LPS) characterized by inflamed LPS cells pretreated with vehicle; positive control (Pacli) cells pretreated with Paclitaxel (30 *μ*M)—a reference proapoptotic drug; and the treatment groups (Dexa) pretreated with Dexamethasone (7 *μ*M) and (Rubiadin-1-methyl ether) pretreated with Rubiadin-1-methyl ether (10, 30, and 100 *μ*M) (*n* = 3/group). In the next step, cells were washed with cold phosphate-buffered saline (PBS; pH 7.6) and centrifuged twice (900 x g, 5 min at 4°C). The supernatant was discarded, and the pellets were suspended in binding buffer (0.1 M Hepes/NaOH (pH 7.4), 1.4 M NaCl, 25 mM CaCl_2_). Next, the cells were stained with annexin V conjugated with fluorescein isothiocyanate (FITC) (BD Biosciences, San Jose, CA, USA) to analyze the apoptosis, following the manufacturer's instructions. The macrophages were differentiated from cell debris based on forward-scattered light (FSC) vs. side-scattered light (SSC) characteristics. The levels of apoptosis and necrosis on the macrophages were analyzed by a FACSVerse® flow cytometer (BD Biosciences, San Jose, CA, USA) using the FACSuite® software, in which 10,000 events were obtained. The results were expressed as percentages.

### 2.4. In Vivo Experiments

#### 2.4.1. Animals

The study was conducted in 4-week-old male Swiss mice weighing 20–25 g. The animals were supplied by the Animal Centre from UFSC and were housed at constant room temperature (20 ± 2°C), under 12 h light/dark cycles, fed with standard mouse chow and water. After the induction with LPS, the mice were euthanized with an overdose of xylazine/ketamine (30 mg/kg and 300 mg/kg, respectively) and the samples were collected for further evaluations. The project was approved by the Committee for Ethics in Animal Research of UFSC (Protocol 6118110417).

#### 2.4.2. LPS-Induced Acute Lung Injury (ALI) Model

The mice were randomly divided into 4 groups (*n* = 6/group): (S) Blank control (saline), represented by healthy animals and pretreated only with 0.9% sterile saline and vehicles (5% Tween 20 and 5% DMSO); (LPS) negative control, also pretreated with the vehicle and induced by LPS (5 mg/kg); (Dexa) positive control, pretreated with Dexamethasone (5 mg/kg, standard treatment animal); and Rubiadin-1-methyl ether treated with Rubiadin-1-methyl ether (3, 10, and 30 mg/kg). All the pretreatments were conducted orally (gavage), and after 1 h, all groups (except the Blank control) were anesthetized with xylazine/ketamine (5 mg/kg and 50 mg/kg) for intranasal LPS instillation (5 mg/kg in 50 *μ*L PBS). After 12 hours, mice were euthanized by an overdose of xylazine/ketamine and bronchoalveolar lavage fluid (BALF) was collected [13]. Immediately after the BALF collection, the total and differential leukocyte counts and exudate concentration (protein content) were performed. Aliquots of BALF were stored in cryotubes and deposited in a freezer at -80°C for further evaluations of myeloperoxidase (MPO) activity, NOx, and pro- and anti-inflammatory cytokine (IL-6, IL-12p70, IL-10, IFN-*γ*, TNF-*α*, and MCP-1) levels.

#### 2.4.3. Total Differential Leukocyte Count and Exudation on BALF

The total leukocyte count in BALF was performed with Türk's solution (1 : 4). A common optical microscope (400x magnification) was used to quantify the cells in a Neubauer chamber. Cytospin slides of BALF were stained with Panoptic® dye for the differential count, which was performed under an oil immersion objective on a common optical microscope. The total and differential leukocyte counts were expressed as cells × 10^5^/mL. The degree of capillarity leakage (exudation) was determined using the Lowry method [14], which quantifies the presence of total proteins in the samples. Bovine serum albumin was used as the standard, and exudation levels were expressed as *μ*g/mL.

#### 2.4.4. Determination of Myeloperoxidase (MPO) Activity in BALF

The MPO assay was conducted according to methods previously described in the literature [15]. Briefly, BALF aliquots were mixed with HTAB (hexadecyltrimethyl ammonium bromide 0.5%), subjected to three vortex/freeze-thaw cycles and centrifuged in refrigerated conditions (40.000 x g, 15 min at 4°C). The supernatants were transferred to a 96-well plate, and MPO activity was performed by colorimetric measurements in an ELISA plate reader at 450 nm by interpolation from MPO (extracted from human leukocyte, Sigma-Aldrich Co.) standard curve (0.07–140 mU/mL). The results were expressed in mU/mL.

#### 2.4.5. Determination of Nitric Oxide Levels (NOx) in BALF

The quantification of nitric oxide (NO) present in each sample was measured indirectly by the presence of its metabolic nitrate (NO_3_-) and nitrite (NO_2_-), following the Griess reaction [16]. BALF aliquots were deproteinized using zinc sulfate (ZnSO4, 20%), with subsequent overnight incubation at -20°C in a freezer, with subsequent addition of sodium hydroxide (NaOH, 2.5 N). The supernatants were transferred to 96-well plates and mixed with saturated vanadium chloride (VCI3) and Griess reagents. The colorimetric measurement was determined at 540 nm using an ELISA plate reader, and the amount of nitric oxide was determined indirectly by interpolation from the nitrite standard curve (0–100 *μ*M). The results were expressed in *μ*M.

#### 2.4.6. Determination of Cytokine Levels in BALF

The pro- and anti-inflammatory cytokine (IL-6, IL-12p70, IL-10, IFN-*γ*, TNF-*α*, and MCP-1) levels present in the BALF were determined by flow cytometry (BD Bioscience FACSVerse® Flow Cytometer), using a commercial kit (Cytometric Bead Array, CBA Mouse inflammation kit). The values were quantified using the FCAP Array® software. The detection limits for IL-6, IL-10, IL-12p70, MCP-1, INF-*γ*, and TNF-*α* were as follows: 5.00 pg/mL, 17.50 pg/mL, 10.70 pg/mL, 52.70 pg/mL, 2.50 pg/mL, and 7.30 pg/mL, respectively. The results were expressed in pg/mL.

#### 2.4.7. Lung Histological Analysis

Mouse lungs were removed 12 h after LPS challenge, washed in PBS, and fixed in formalin solution 10% (*v*/*v*). The tissues were dehydrated in ethanol solutions, followed by xylene, then included in paraffin and sliced into 4 *μ*m sections (LEICA-Instruments® CM3050, Nussloch, Baden-Württemberg, Germany). Further, the slices were stained with hematoxylin-eosin and analyzed under light microscopy (200x). The lung damage was graded taking into account the degree of parenchymal distortion in the alveolar tissue. The scores used were as follows: 0—normal, 1—in-creased thickness in <50% of interalveolar septa (IAS) due to edema and/or neutrophil infiltration, 2—increased thickness in >50% of IAS, 3—increased thickness in >50% of IAS and the presence of neutrophils within the alveolar space, and 4—consolidated infiltration of neutrophils with distortion of normal alveolar architecture. The mean score was reported for each microscope section.

### 2.5. Statistical Analysis

The results were analyzed by GraphPad Prism® version 7.0 (San Diego, California, USA) and IBM SPSS Statistics 22. Parametric experimental results were expressed as mean ± standard error of the mean (S.E.M.). Data residuals were analyzed for normality using the Shapiro-Wilk test and homoscedasticity using the Bartlett test. For the parametric homoscedastic data, one-way ANOVA followed by Tukey's post hoc test was used. Parametric heteroscedastic data were analyzed by one-way ANOVA-Welch followed by the Games-Howell post hoc test. Significance was set at *P* < 0.05.

## 3. Results

### 3.1. In Vitro Experiments

#### 3.1.1. Cytotoxicity of Rubiadin-1-methyl Ether

**Figure 2:**
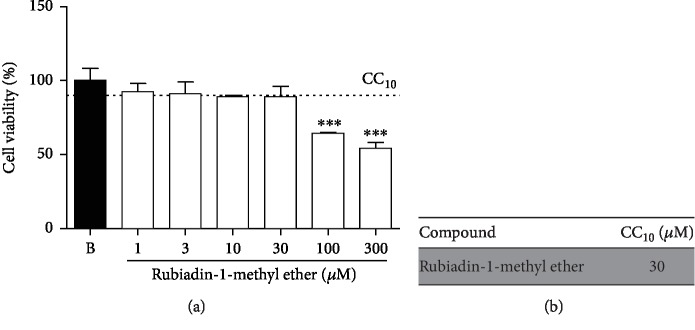
Evaluation of Rubiadin-1-methyl ether cytotoxicity (RAW 264.7) (a) and calculated CC_10_ (b) for this compound. B: cells pretreated with vehicle and stimulated with PBS; Rubiadin-1-methyl ether: cells pretreated with Rubiadin-1-methyl ether at doses at 1 to 300 *μ*M. Each bar represents the average survival of the macrophages in independent experiments ± S.D. (n = 3). ^∗∗∗^*P* < 0.001.

Rubiadin-1-methyl ether did not exhibit significant toxicity, since this effect was observed only in high concentrations (up to 100 *μ*M) (Figure 2(a)). Moreover, it was possible to determine the CC_10_ from this compound—a protocol used to ensure a 90% safe viability during the experiments (Figure 2(b)).

#### 3.1.2. Effect of the Rubiadin-1-methyl Ether on NOx Levels

**Figure 3:**
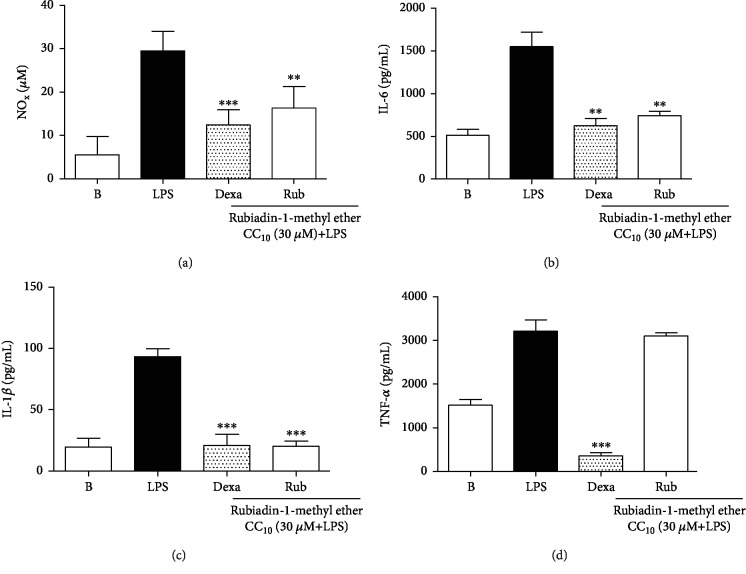
Effect of Rubiadin-1-methyl ether at 30 *μ*M dose on NOx (a), IL-6 (b), IL-1*β* (c), and TNF-*α* secretion (d) in RAW 264.7 macrophages stimulated with LPS. B: cells pretreated with vehicle and stimulated with PBS; LPS: cells stimulated with LPS (1 *μ*g/mL); Dexa: cells pretreated with Dexamethasone (7 *μ*M) and stimulated with LPS (1 *μ*g/mL); Rub: cells pretreated with Rubiadin-1-methyl ether at 30 *μ*M and stimulated with LPS (1 *μ*g/mL). The results were expressed as the mean ± S.D.; *n* = 3; ^∗∗^*P* < 0.01 and ^∗∗∗^*P* < 0.001 compared with the LPS group.

Next, NOx assay was performed using the CC_10_ value (30 *μ*M). In these experiments, Rubiadin-1-methyl ether was able to significantly inhibit NOx production (% of inhibition: 44.7 ± 9.6) (*P* < 0.01) (Figure 3(a)). At the same manner, Dexamethasone also inhibited the synthesis of this inflammatory mediator (% of inhibition: 58.1 ± 4.9) (*P* < 0.001) (Figure 3(a)).

#### 3.1.3. Effect of the Rubiadin-1-methyl Ether on Proinflammatory Cytokines (IL-6, IL-1*β*, and TNF-*α*)

In these experiments, Rubiadin-1-methyl ether at its CC_10_ dose significantly inhibited the production of IL-6 and IL-1*β*. The effect on IL-6 levels produced a significant inhibition (% of inhibition: 52.1 ± 3.2) (*P* < 0.01) (Figure 3(b)). Likewise, Rubiadin-1-methyl ether significantly inhibited the IL-1*β* levels (% of inhibition: 78.0 ± 4.1) (*P* < 0.001) (Figure 3(c)). On the other hand, this compound was not able to inhibit the TNF-*α* levels (*P* > 0.05) (Figure 3(d)). As expected, Dexamethasone significantly reduced the levels of all cytokine studied (% of inhibition: 59.7 ± 5.4, 77.4 ± 9.3, and 88.66 ± 1.6) (*P* < 0.01) (Figures 3(b)–3(d)).

#### 3.1.4. Effect of the Rubiadin-1-methyl Ether on Macrophage Apoptosis

**Figure 4:**
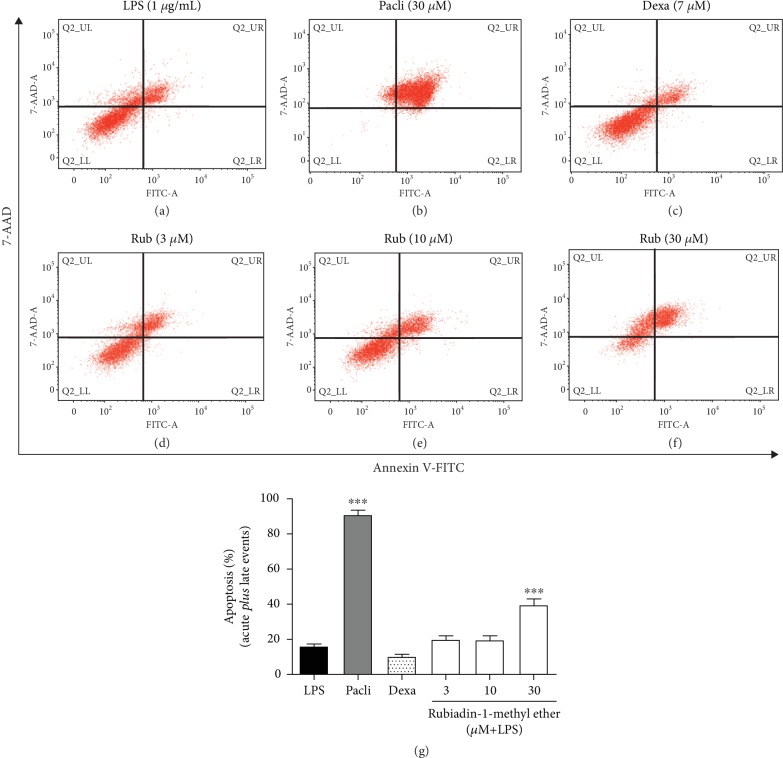
Flow cytometry dot plots representing the effect of Rubiadin-1-methyl ether on RAW 264.7 macrophage apoptosis (acute *plus* late events). LPS: cells stimulated with LPS (1 *μ*g/mL) (a); Paclitaxel: cells pretreated with Paclitaxel at 30 *μ*M before LPS administration (b); Dexa: cells pretreated with Dexamethasone (7 *μ*M) 30 min before LPS administration (c); and Rub: cells pretreated with Rubiadin-1-methyl ether at 3 *μ*M (d), 10 *μ*M (e), and 30 *μ*M (f). Results presented at graphic mode (g) and expressed as the mean ± S.D.; *n* = 3; ^∗∗∗^*P* < 0.001 compared with the LPS group.

Due to the significant correlation between the inflammatory process and the reduction on leukocyte apoptosis [17], we evaluated the possible action of Rubiadin-1-methyl ether on this parameter. Our results demonstrated that Rubiadin-1-methyl ether was able to reverse the decrease in apoptosis rate produced by the inflammatory reaction when used at 30 *μ*M (% of increase: 149.7 ± 31.7) (*P* < 0.001) (Figures 4(f) and 4(g)). Dexamethasone also was not able to reverse this proinflammatory finding, when used at the tested concentration (*P* > 0.05) (Figures 4(c) and 4(g)).

### 3.2. In Vivo Experiments

#### 3.2.1. Effect of the Rubiadin-1-methyl Ether on Cell Migration and Exudation

**Figure 5:**
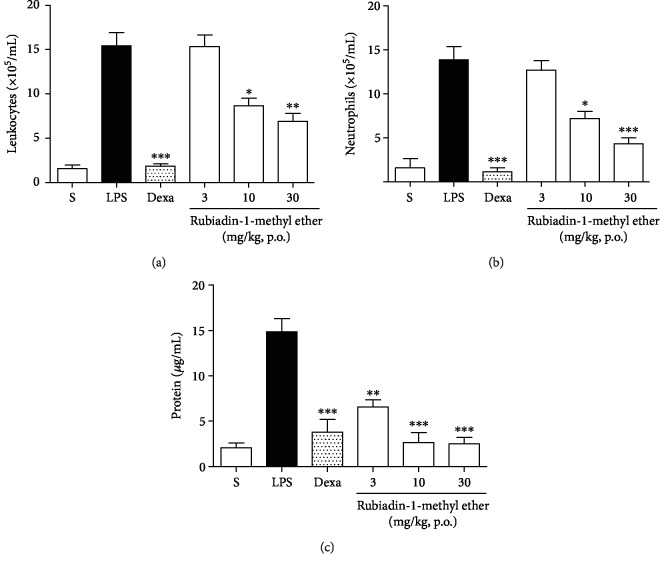
Effect of Rubiadin-1-methyl ether on total leukocyte counts (a), neutrophil counts (b), and exudation (c). S: healthy animals only treated with 0.9% sterile saline and vehicle (1% DMSO); LPS: (negative control) inflamed animals only stimulated with LPS (1 *μ*g/mL) at intranasal route (i.n.); Dexa: (positive control) animals pretreated with Dexamethasone (5 mg/kg, p.o.) 1 h before LPS administration; Rubiadin-1-methyl ether: animals pretreated with Rubiadin-1-methyl ether at doses of 3, 10, and 30 mg/kg (p.o.) 1 h before LPS administration. The results were expressed as the mean ± S.D.; *n* = 6; ^∗^*P* < 0.05, ^∗∗^*P* < 0.01, and ^∗∗∗^*P* < 0.001 compared with the LPS group.

Rubiadin-1-methyl ether significantly decreased the total leukocyte count, showing a substantial inhibition when administrated at 10 and 30 mg/kg (% of inhibition: 43.6 ± 5.4 and 55.3 ± 5.8), respectively (*P* < 0.001) (Figure 5(a)). This inhibition was due to the ability of this compound in reducing the neutrophil migration (% of inhibition: 48.1 ± 5.6 and 68.8 ± 4.7), respectively (*P* < 0.001) (Figure 5(b)). Furthermore, Rubiadin-1-methyl ether demonstrated the capacity to reduce exudate formation in the lungs. This decrease was observed in all tested doses (3, 10, and 30 mg/kg) (% of inhibition: 55.5 ± 4.9, 81.9 ± 6.9, and 82.9 ± 45), respectively (*P* < 0.01) (Figure 5(c)).

#### 3.2.2. Effect of the Rubiadin-1-methyl Ether on Nitric Oxide Levels (NOx) and Myeloperoxidase (MPO) Activity

**Figure 6:**
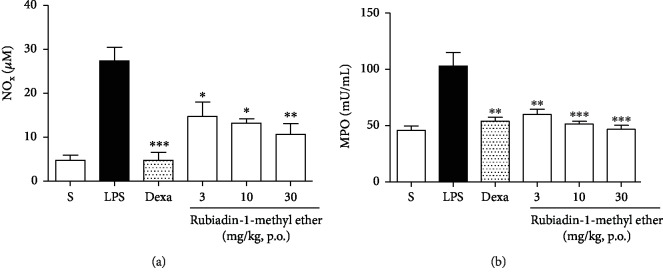
Measurement of NO levels and MPO activity. Effect of Rubiadin-1-methyl ether on measurement of NO levels (a) and MPO activity (b). S: healthy animals only treated with 0.9% sterile saline and vehicle (1% DMSO); LPS: (negative control) inflamed animals only stimulated with LPS (1 *μ*g/mL) at intranasal route (i.n.).; Dexa: (positive control) animals pretreated with Dexamethasone (5 mg/kg, p.o.) 1 h before LPS administration; Rubiadin-1-methyl ether: animals pretreated with Rubiadin-1-methyl ether at doses at 3, 10, and 30 mg/kg (p.o.) 1 h before LPS administration. The results were expressed as the mean ± S.D.; *n* = 6; ^∗^*P* < 0.05, ^∗∗^*P* < 0.01, and ^∗∗∗^*P* < 0.001 compared with the LPS group.

Similar, to the *in vitro* experiments, Rubiadin-1-methyl ether also demonstrated effectiveness to reduce nitric oxide (NOx) metabolites, as well as MPO activity in the BALF. The NOx levels were reduced in all tested doses (3, 10, and 30 mg/kg) (% of inhibition: 46.3 ± 12.0, 51.8 ± 3.7, and 60.1 ± 8.7), respectively (*P* < 0.05) (Figure 6(a)). With the same pattern, this compound reduced the MPO activity in all tested doses (% of inhibition: 41.8 ± 4.5, 50.1 ± 2.5, and 54.7 ± 3.5), respectively (*P* < 0.01) (Figure 6(b)).

#### 3.2.3. Effect of the Rubiadin-1-methyl Ether on Pro- and Anti-Inflammatory Cytokines

Rubiadin-1-methyl ether demonstrated a significant capacity on the decrease of the levels of all proinflammatory cytokines studied at least, in one of the tested doses. IL-12p70 and IL-6 demonstrated a significant inhibition in all tested doses (% of inhibition: 3 mg/kg: 37.1 ± 11.3, 10 mg/kg: 47.4 ± 13.9, and 30 mg/kg: 79.5 ± 8.2) and (% of inhibition: 3 mg/kg: 33.7 ± 10.5, 10 mg/kg: 49 ± 6.9, and 30 mg/kg: 79.5 ± 6.8), respectively (*P* < 0.05) (Table 1). INF-*γ* demonstrated a significant reduction when Rubiadin-1-methyl ether was used at 10 and 30 mg/kg (% of inhibition: 10 mg/kg: 64.5 ± 5.6 and 30 mg/kg: 88.4 ± 1.8) (*P* < 0.01) (Table 1), while the chemokine MCP-1 showed a significant reduction only when the compound was used at the highest tested dose (% of inhibition: 30 mg/kg: 82.6 ± 7.2) (*P* < 0.01) (Table 1). In contrast with the results obtained *in vitro*, Rubiadin-1-methyl ether was able to reduce TNF-*α* production, at a significant manner in an *in vivo* model (% of inhibition: 10 mg/kg: 27.7 ± 6.6 and 30 mg/kg: 69.2 ± 11.4) (*P* < 0.05) (Table 1). Furthermore, this compound caused a significant increase in the production of IL-10 when administrated at 10 and 30 mg/kg (% of increase: 10 mg/kg: 324.8 ± 67 and 30 mg/kg: 360.5 ± 74.8) (*P* < 0.001) (Table 1).

#### 3.2.4. Effect of the Rubiadin-1-methyl Ether on Lung Histological Architecture

**Figure 7:**
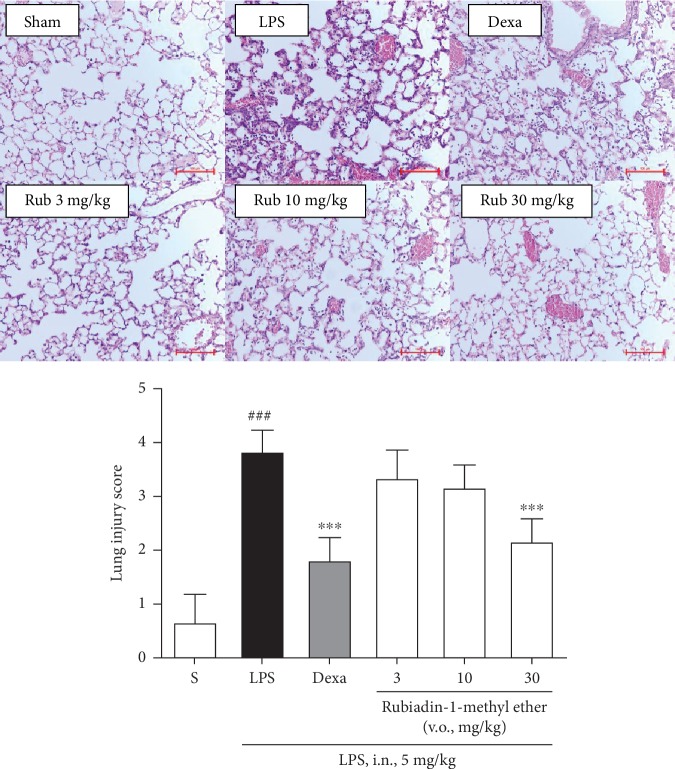
Effect of Rubiadin-1-methyl ether on lung architecture in LPS-induced acute lung injury mice. Sham: animals treated with vehicle per oral 1 h before intranasal instillation with sterile saline; LPS: (negative control) animals treated with vehicle per oral 1 h before intranasal instillation with sterile LPS; Dexa: (positive control) animals treated with dexamethasone per oral (5 mg/kg) 1 h before intranasal instillation with sterile LPS; Rub (3, 10, and 30 mg/kg): animals treated with Rubiadin-1-methyl ether per oral (3, 10, and 30 mg/kg) 1 h before intranasal instillation with sterile LPS. Each group represents the mean ± standard error of the mean; *n* = 6/group. ^###^*P* < 0.001 compared to the Sham group. ^∗∗∗^*P* < 0.001 compared to the LPS group.

The acute lung injury induction by LPS intranasal instillation provided a massive leukocyte infiltration with a consequent loss of lung architecture. The pretreatment with Rubiadin-1-methyl ether reduced this lung damage with a statistical significance, at the dose of 30 mg/kg (% inhibition: 43.4 ± 4.3) (Figure 7). Dexamethasone treatment also significantly reduced the histological parameters studied (% inhibition: 47.7 ± 6.7) (*P* < 0.001) (Figure 7).

## 4. Discussion

Despite intense efforts to use approved drugs in the treatment of critical medical conditions like ALI, nowadays, there is no medicine that can significantly improve the poor prognosis of this condition [18, 19]. However, the use of glucocorticoids as an adjuvant therapy to prevent the progression of ALI to circulatory and metabolic dysfunction is an approach used worldwide [20, 21]. Studies have therefore focused on the search for a compound capable of reversing this clinical situation.

Natural products are the most versatile source of new compounds with biologicals effects, since the majority of drugs currently in use are derived or synthesized from natural molecules and their products [22]. Anthraquinones, a natural class of compounds used as laxative, antimalarial [23, 24], anticancer [25], and antiviral [26] treatments, recently demonstrated anti-inflammatory action by interfering in the intracellular signaling related to the activation of STAT proteins [27]. It is not unexpected that signaling conducted by various STATs, particularly STAT3, will be closely interconnected with NF-*κ*B signaling—the nuclear factor responsible for managing the production of proinflammatory mediators in ALI, sepsis, and systemic shock [28–30].

Among the main mediators involved in the development of ALI and systemic shock initiated by LPS [31], NO deserves a highlighted role, as it is the main factor responsible for producing uncontrolled systemic vasodilatation, leading to intravascular coagulation and, in critical cases, culminating in organ failure [32, 33]. NO production, in the inflammatory context, is mainly conducted by iNOS (inducible nitric oxide synthase) that produces 100 to 1000x more NO than the constitutive isoforms [34]. In our experiments, Rubiadin-1-methyl ether, a major anthraquinone isolated from *Pentas schimperi*, showed significant capacity to inhibit the production of this mediator in both models—*in vitro* and *in vivo*. A similar effect was observed in the *in vitro* experiments conducted by Alves and coworkers, who demonstrated an inhibitory effect of mitoxantrone (an anthraquinone used in anticancer therapy) on NO levels, using a model of J774 macrophages stimulated by LPS [35]. More recently, Vien et al. showed, through their research, the inhibitory effects of seven different anthraquinones on NO levels. This effect was attributed to the ability of these compounds to inhibit iNOS expression in the same *in vitro* inflammatory model as that used in our experiments [36].

Besides regulating nitric oxide production, reducing the progression to ALI from systemic shock, chemokines and pro- and anti-inflammatory cytokines like TNF-*α*, IL-6, IL-12, IFN-*γ*, MCP-1, and IL-10 are important mediators involved in the establishment of ALI critical findings [37, 38]. The excessive production of proinflammatory cytokines and chemokines promotes the expression of tissue growth factors produced by activated macrophages and leads to a coagulation disorder culminating in multiple organ failure [39]. In this context, our results demonstrated that Rubiadin-1-methyl ether tested *in vitro*, as well as *in vivo*, was able to reduce the production of proinflammatory cytokines and chemokines induced by LPS. Moreover, this compound was able to increase the production of IL-10, a cytokine with anti-inflammatory profile. Corroborating with our results, other authors have also observed the same inhibitory effects related to different anthraquinone compounds. Likewise, the experiments conducted by Hu and coworkers demonstrated an inhibitory action of aloe emodin from rhubarb on NOx levels, as well on proinflammatory cytokines (IL-6 and IL-1*β*) [40]. Another study, conducted by Wu et al., demonstrated a significant reduction on the production of proinflammatory cytokines when U937 and RAW 264.7 cells were pretreated with 6-hydroxyrubiadin before stimulation with phorbol myristate acetate (PMA) and LPS, respectively [41]. These authors also proved that 6-hydroxyrubiadin had important *in vivo* effects, demonstrating that this anthraquinone maintains an inhibitory effect on proinflammatory cytokines in mice pretreated before LPS administration, significantly attenuating the severity of LPS-induced ALI [41].

In addition, the ability of Rubiadin-1-methyl ether to increase the secretion of IL-10 deserves to be highlighted, since this effect can be considered a highly desirable regulatory mechanism in inflammatory conditions. Recently, IL-10 has been shown to be an important cytokine responsible for shifting the macrophage function, promoting the modulation of cellular metabolism, and resulting in the immunomodulation of the inflammatory reaction [42]. This cytokine is emphasized in the inflammatory process due its regulatory character, balancing and suppressing the expression of proinflammatory cytokines, such as IL-6, IL-1*β*, and TNF-*α* [43]. Similar results were obtained by Meng et al. who demonstrated that *Rheum tanguticum Maxim*. ex Balf. (*Rt*), a traditional Tibetan medicine rich in anthraquinones, had anti-inflammatory effects by inhibiting the secretion of proinflammatory cytokines and NO. Furthermore, an immunomodulatory character was revealed based on the ability of this compound to stimulate the secretion of IL-10 on primary microglia cells from the cerebral cortex of C57BL/6 mice [44].

In conjunction with the reduction in plasma extravasation, the reduction in neutrophil recruitment is one of the steps necessary to reconstitute tissue homeostasis on ALI, followed by a return to the normal rate of inflammatory cell apoptosis [45, 46]. In this sense, our *in vivo* experiments demonstrated that Rubiadin-1-methyl ether had the ability to reduce fluid leakage and leukocyte influx in the BALF. This effect can be attributed to its capacity in reduce neutrophil migration and activation (MPO). Moreover, our *in vitro* assays demonstrated that macrophages reestablished the normal apoptosis rate when pretreated with Rubiadin-1-methyl ether before LPS administration. Other researchers have already attributed this effect to anthraquinone compounds, e.g., Wang and coworkers, who demonstrated, using an elegant experimental model, that emodin was able to reestablish the apoptosis rate on neutrophils isolated from rats with severe acute pancreatitis induced by sodium taurocholate [47]. In fact, contact with apoptotic neutrophils may shift the profile of proinflammation-activated macrophages to an anti-inflammatory state [48], as the interplay between apoptotic cells and mononuclear phagocytes suppresses the inflammatory responses and facilitates apoptotic cell clearance [49]. Taken together, the findings obtained in our experiments allowed us to hypothesize that the compound studied probably reduces the need for the macrophages to sustain a proinflammatory phenotype (M1), at least in the LPS models used.

## 5. Conclusion

In summary, we demonstrated that the compound Rubiadin-1-methyl ether has significant anti-inflammatory activity, showing an immunomodulatory profile. This compound may therefore be a potential candidate for the development of treatments for inflammatory conditions in which LPS is closely related, such as acute lung injury (ALI). However, further studies are needed to accurately elucidate the exact mechanism of action in the LPS cell pathway.

## Figures and Tables

**Table 1:** Effects of Rubiadin-1-methyl ether on pro- and anti-inflammatory cytokine levels in LPS-induced acute lung injury (ALI) in mice.

Cytokines	TNF-*α* (pg/mL)	IL-6 (pg/mL)	MCP-1 (pg/mL)	IL-12p70 (pg/mL)	INF-*γ* (pg/mL)	IL-10 (pg/mL)
S	136.0 ± 17.3	6.9 ± 0.8	21.9 ± 7.1	2.1 ± 0.3	2.8 ± 0.9	9.7 ± 1.0
LPS	7102.0 ± 645.0	4481 ± 153.8	609.0 ± 44.5	48.8 ± 3.8	399.8 ± 80.8	3.7 ± 0.7
Dexa	298.5±76.0^∗∗∗^	270.0±27.0^∗∗∗^	20.9±5.2^∗∗∗^	4.8±1.2^∗∗∗^	2.7±0.8^∗∗∗^	9.0 ± 1.9^∗^
Rub (3)	6229.0 ± 986.2	2968.0 ± 469.7^∗^	507.3 ± 158.5	30.7 ± 5.5^∗^	259.3 ± 56.3	3.3 ± 0.9
Rub (10)	5162.0 ± 471.6^∗^	2285.0±311.9^∗∗∗^	444.3 ± 65.9	25.7 ± 6.8^∗^	141.8±22.5^∗∗^	15.8±2.5^∗∗∗^
Rub (30)	2188.0±812.4^∗∗∗^	921.1±308.3^∗∗∗^	105.9±43.9^∗∗^	10.0+4.0^∗∗∗^	46.3±7.2^∗∗∗^	17.1±2.8^∗∗∗^

S: health animals treated with vehicle per oral 0.5 h before intranasal instillation with sterile saline; LPS: negative control, also pretreated with vehicle and induced by LPS (5 mg/kg); Dexa: positive control, pretreated with Dexamethasone (5 mg/kg) per oral administrated 0.5 h before acute lung injury induction; Rub: treatment with Rubiadin-1-methyl ether (3, 10, and 30 mg/kg) administered per oral 0.5 h before LPS-induced acute lung injury. Each group represents the mean ± SEM; N = 5 animals; ^∗^*P* < 0.05; ^∗∗^*P* < 0.01; ^∗∗∗^*P* < 0.001.

## Data Availability

No data were used to support this study.
